# Multivariate Patterns of Cognitive and Socio-Cognitive Deficits in Schizophrenia, Bipolar Disorder, and related risk

**DOI:** 10.1192/j.eurpsy.2025.104

**Published:** 2025-08-26

**Authors:** A. Raio, L. A. Antonucci

**Affiliations:** Department of Translational Biomedicine and Neuroscience, University of Bari, Bari, Italy

## Abstract

**Abstract:**

Alterations in cognition and social cognition in bipolar disorder and schizophrenia have been largely documented. However, to which extent these alterations overlap between the disorders and how they are relevant to early stages as well as to risk conditions remains unclear. To shed light on this topic, 59 patients with Bipolar Disorder (BD), 118 patients with schizophrenia (SCZ), two independent cohorts of Healthy Controls (HC1=95, HC2=195), as well as individuals at Clinical High Risk (CHR=35) and at First Episode of Psychosis (FEP=29) were characterized for a series of cognitive and socio-cognitive features, which were entered in a machine learning analysis as a cognitive, a socio-cognitive, and a combined cognitive and socio-cognitive (stacking) classifier. Such classifiers were probed to discriminate at the single subject level BD vs. HC1 and SCZ vs. HC2. Then, those with the greatest diagnostic power in categorizing BD vs. HC1 were challenged to predict discrimination between SCZ vs. HC2, and vice-versa. Thus, decision scores for such models were compared with those obtained when they were applied to FEP and CHR. Results indicated that stacking classifiers were the best in discriminating HC1 vs. BD (Balanced Accuracy - BAC = 80%) and HC2-SCZ (BAC = 84%). Furthermore, the HC1-BD staking classifier successfully discriminated HC2 from SCZ (BAC=77.4%), and vice-versa (BAC=83.1%). Decision scores for SCZ and BD overlapped with those obtained when stacking models were applied to FEP, identifying a “patient-like” pattern. Differently, when such combined signatures were applied to CHR individuals, they were classified neither as patients nor as HC. Findings suggest an overall overlap of cognitive and socio-cognitive anomalies classifying schizophrenia and bipolar disorder, which is also relevant to early stages of disease. In this general context, disease-specific core abnormalities characterize SCZ and BD. Personalized rehabilitative programs may be oriented to primarily manage disease-related cognitive and socio-cognitive “hub” alterations, but always within a broader assessment.

**Disclosure of Interest:**

None Declared

